# Morphometry of the suprascapular notch: correlation with scapular dimensions and clinical relevance

**DOI:** 10.1186/1471-2474-14-172

**Published:** 2013-05-24

**Authors:** Paolo Albino, Stefano Carbone, Vittorio Candela, Valerio Arceri, Anna Rita Vestri, Stefano Gumina

**Affiliations:** 1 Department of Orthopaedics and Traumatology, University of Rome “Sapienza”, Piazzale Aldo Moro 3, Rome, 00185, Italy; 2 Department of Public Health and Infectious Diseases, University of Rome “Sapienza”, Rome, Italy

**Keywords:** Suprascapular notch, Suprascapular nerve, Suprascapular nerve entrapment, Scapula, Anatomic classification

## Abstract

**Background:**

Better knowledge of the suprascapular notch anatomy may help to prevent and to assess more accurately suprascapular nerve entrapment syndrome. Our purposes were to verify the reliability of the existing data, to assess the differences between the two genders, to verify the correlation between the dimensions of the scapula and the suprascapular notch, and to investigate the relationship between the suprascapular notch and the postero-superior limit of the safe zone for the suprascapular nerve.

**Methods:**

We examined 500 dried scapulae, measuring seven distances related to the scapular body and suprascapular notch; they were also catalogued according to gender, age and side. Suprascapular notch was classified in accordance with Rengachary’s method. For each class, we also took into consideration the width/depth ratio. Furthermore, Pearson's correlation was calculated.

**Results:**

The frequencies were: Type I 12.4%, Type II 19.8%, Type III 22.8%, Type IV 31.1%, Type V 10.2%, Type VI 3.6%. Width and depth did not demonstrate a statistical significant difference when analyzed according to gender and side; however, a significant difference was found between the depth means elaborated according to median age (73 y.o.). Correlation indexes were weak or not statistically significant. The differences among the postero-superior limits of the safe zone in the six types of notches was not statistically significant.

**Conclusions:**

Patient’s characteristics (gender, age and scapular dimensions) are not related to the characteristics of the suprascapular notch (dimensions and Type); our data suggest that the entrapment syndrome is more likely to be associated with a Type III notch because of its specific features.

## Background

The suprascapular notch (SN) serves as passage to the suprascapular nerve (SSN) and it is converted into a foramen by the superior transverse scapular ligament [1]. The first description of suprascapular nerve entrapment syndrome at the site of the suprascapular notch was made by Kopell and Thompson [2]. Over the years, many studies have investigated and have identified the pathologic factors related to this syndrome. The following proved to be involved in the aetiology as well as iatrogenic lesions during open or arthroscopic surgical procedures [3-5]: anterior shoulder dislocation [6]; injury from direct trauma [6]; ganglion cysts [7]; synovial and Ewing’s sarcomas [8]; and chondrosarcoma and lipoma [8].

**Figure 1:**
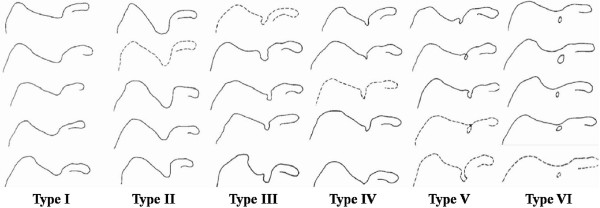
**Renganchary's classification.** Schematic illustration.

Anatomical variations [3,9,10] and the anomalous or ossified superior transverse scapular ligament [9,11,12] are also considered to be risk factors for suprascapular neuropathy. In previous studies, different types of SN were identified [3,9,10]. Rengachary [3] distinguished VI types of SN basing the classification on morphologic and geometric features (Figure 1).

In Type I the superior border of the scapula present a depression from the medial superior angle to the base of the coracoid process; Type II is described as a wide blunded “V”-shaped notch, occupying nearly a third of the superior border with the widest point along the superior border. Type III is a simmetric and “U”-shaped notch while Type IV is described as a very small “V”-shaped notch, frequently presenting a shallow groove near the notch; Type V is similar to type III, with partial ossification of the medial border of the notch and with the minimal diameter along the superior border of the scapula. Type VI is described as a bony foramen with a completely ossified STSL.

The suprascapular nerve may be accidentally injured in many surgical procedures, for example blind drilling during arthroscopic Bankart [4] and SLAP repair [13-15] advancement of rotator cuff during the repair of massive retracted rotator cuff tears [5,16-23], arthroscopic anterior or double interval slide [5] and also during the decompression of suprascapular nerve entrapment [3,24]. In order to avoid this complication, previous anatomical studies analyzed the “safe zone” [4,13,25-27], considered as an area within which is possible to avoid iatrogenic lesions of the suprascapular nerve. The safe zone presents two safe limits: the posterior represented as the distance measured from the supraglenoid tubercle to the scapular notch and the posterosuperior represented as the distance measured from the midline of the posterior glenoid rim to the base of the scapular spine [27].

We aimed to verify the reliability of the existing data concerning the anatomy of the SN, by analyzing a considerable number of dried scapulae and comparing our results with literature. Measurements were performed in order to assess the differences between scapulae in the two genders and within each individual subject, by comparing the dimensions of the right and left scapula of the same subject. Furthermore, we assessed potential correlations between the dimensions of the SN [3] and the major dimensions of the scapula [27]. We also evaluated variations of the dimensions and of the area among the different types of the SN, as well as the relationship between different types and the posterosuperior limit of the safe zone for the suprascapular nerve [27].

## Methods

We examined 500 dried scapulae belonging to the anatomical collection of the Department of Anatomy from two Universities (53 + 59), the bone collection of the municipality of Rome (70), and a southern Italian region (318). Our research was performed in compliance with the Helsinki Declaration.

The gender and ages of 155 donors were known. Among the donors, we found 131 complete skeletons (67 males, 64 females) while in the remaining 24 (5 males, 19 females) one of the two scapulae was missing. Therefore, there were a total of 147 scapulae belonging to females and 139 to males. An additional 15 complete skeletons, whose gender and age were unknown, were used. The age at the time of death ranged from 22 to 108 years (average age: 71).

**Figure 2:**
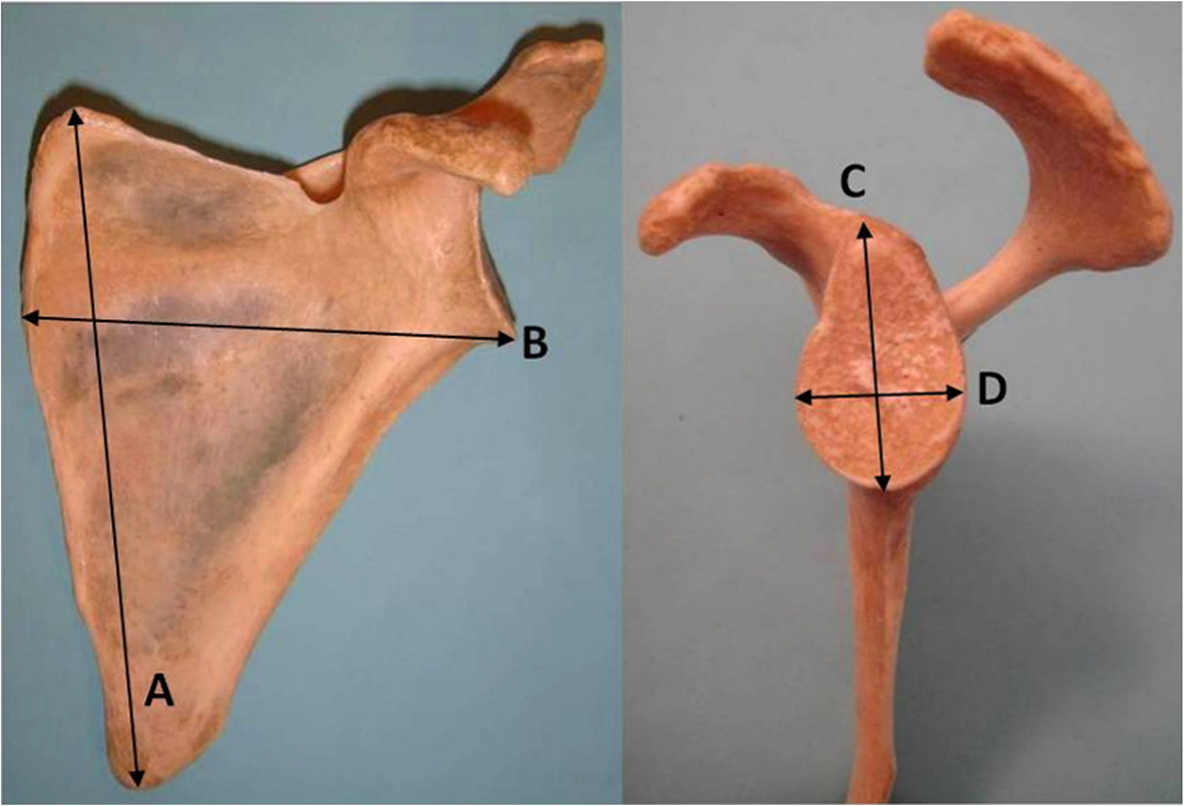
**Photograph of a left scapula.** Anterior view: **A**= major longitudinal axis of the scapular body; **B**= major transversal axis of the scapular body. Lateral view: **C**= major longitudinal axis of the glenoid fossa; **D**= major transversal axis of the glenoid fossa.

**Figure 3:**
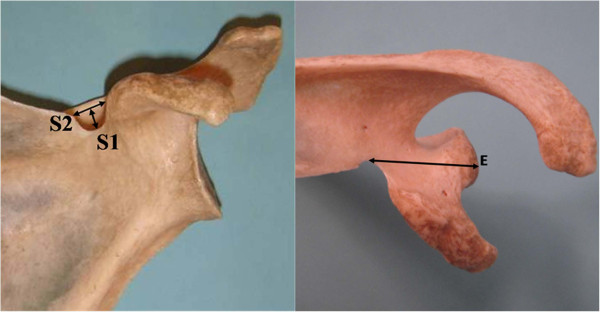
**Photograph of a left scapula.** Anterior view: S1= depth of the suprascapular notch; S2= width of the suprascapular notch. Superior view: E= postero-superior limit of the safe zone.

A researcher particularly skilled in shoulder disorders, measured six distances for each scapula: (i) the major longitudinal axis of the scapular body, measured from the medial angle to the inferior angle of the scapula (A axis) [27]; (ii) the major transversal axis of the scapular body, measured from the lowest point of the glenoid to the vertebral border of the scapula at the level of the smooth surface over which the trapezius glides (B axis) [27]; (iii) the major longitudinal axis of the glenoid fossa, measured from the supraglenoid tubercle to the lowest point of the glenoid (distance C) [27]; (iv) the major transversal axis of the glenoid fossa, measured at its widest distance from the midline of the anterior to the midline of the posterior glenoid rim (distance D) [27] (Figure 2); (v) the distance from the supraglenoid tubercle to the scapular notch (distance E in Figure 3) [27]; and (vi) the depth of the suprascapular notch (S_1_ in Figure 3), measured as the maximal vertical distance from the superior border to lowest point of the suprascapular notch and (vii) the line representing the width of the suprascapular notch (S_2_ in Figure 3), measured along the superior border from the medial to the lateral margin of the notch [3]. We decided to investigate the dimensions of the SN using only S_1_ and S_2_ which proved to be the most helpful ones in Rengachary’s analysis [3]; only the width of Type VI notches was measured as the diameter at the widest point (D_2_ diameter in Rengachary’s method). As a matter of fact, in Type VI notches the diameter measured along the superior border, from the medial to the lateral margin, is 0 by definition.

The shape of the scapular notch was also classified. We decided to apply Renganchary’s system [3] since it is a simple, reproducible and objective method of classification. In addition, it has already been used in several studies and it allowed us to compare the results.

For each class, we analyzed the width/depth ratio expressing the area occupied by the SN.

All measurements were performed twice with a vernier caliper.

### Statistical analysis

Continuous variables were reported by mean, standard deviation, median. Categorical variable were expressed in numerical (count) and percentage terms. To assess differences between groups we used ANOVA. We also calculated the correlation with Pearson’s r. We also estimated the intra-class correlation coefficient (ICC) in order to assess potential statistical bias related to the measuring method.

A value of p <0.05 was considered statistically significant. All analyses were performed using SPSS v.18.

## Results

Measurements related to major dimensions of the scapula, to the posterosuperior limit of the safe zone for the suprascapular nerve and to the dimensions of the SN (width and depth) are reported in Table 1.

**Table 1:** Descriptive analysis of the major dimensions of the scapula, glenoid fossa and scapular notch

	**Mean**	**Median**	**Mode**	**Std. deviation**	**Minimum**	**Maximum**
**Age**	71.3	73.5	77	15.1	22	108
**A axis (cm)**	14.1	14.0	13.5	1.38	10.9	18.8
**B**	10.3	10.3	9.6	0.90	7.8	12.9
**C**	3.6	3.6	3.6	0.36	2.3	5.2
**D**	2.6	2.6	2.4	0.32	1.9	4.3
**Distance E (cm)**	3.1	3.1	3.2	0.32	2.1	4.1
**Depth SN (cm) width**	0.5	0.5	0.5	0.23	0.1	1.9
	0.9	0.9	0.7	0.36	0.1	3.1

*SN* suprascapular notch. *A Axis*= major longitudinal axis of the scapular body; *B Axis*= major transversal axis of the scapular body; *C Axis*= major longitudinal axis of the glenoid fossa; *D Axis*= major transversal axis of the glenoid fossa; *Distance E*= postero-superior limit of the safe zone.

In examining frequencies and percentages of SN types, there is an important prevalence of Type IV among the whole population (details in Table 2).

**Table 2:** Frequencies and percentages of the suprascapular notch types

		**Frequency**	**Percent (%)**
**Scapular notch type**	**I**	62	12.4
	**II**	99	19.8
	**III**	114	22.8
	**IV**	155	31.1
	**V**	51	10.2
	**VI**	18	3.6
**Total**		500	100

Both width and depth of the SN did not show a statistical significant difference when elaborated according to gender (mean width in males: 0.93 cm, mean width in females: 0.94 cm; mean depth in males: 0.61 cm, mean depth in females: 0.58 cm) and side (mean width in right scapulae: 0.96 cm, mean width in left scapulae: 0.95 cm; mean depth in right scapulae: 0.59 cm, mean depth in left scapulae: 0.56 cm).

We found a statistically significant difference (p=0.033) among the mean depths elaborated according to the median age of our sample; subjects over 73 showed a deeper notch (mean: 0.64 cm; 95% IC: 0.59-0.69 cm) than those ≤ 73 (mean: 0.56 cm; 95% IC: 0.51-0.61 cm).

The Pearson correlation indexes, which assess the correlation between A, B, C, D, E distances and the dimensions of the SN (width and depth) are explained in Table 3. We found a weak but statistically significant correlation between the depth of the SN and the major dimensions of the glenoid fossa (C and D axes) as well as between the width of the SN and distance E.

**Table 3:** Correlation indexes between the dimensions of the scapula and the dimensions of the scapular notch

	**A axis**	**B axis**	**C axis**	**D axis**	**Distance E**
**Depth SN**					
**Pearson’s**	0.079	0.084	0.090*	0.115*	0.000
**correlation**					
**Sig. (2-tailed)**	0.084	0.066	0.045	0.010	0.999
**Width SN**					
**Pearson’s**	0.036	0.051	0.028	−0.016	0.183*
**correlation**					
**Sig. (2-tailed)**	0.430	0.267	0.537	0.722	0.000

* statistical significant (p<0,005) correlation indexes. *SN* suprascapular notch.

*A Axis*= major longitudinal axis of the scapular body; *B Axis*= major transversal axis of the scapular body; *C Axis*= major longitudinal axis of the glenoid fossa; *D Axis*= major transversal axis of the glenoid fossa; *Distance E*= postero-superior limit of the safe zone.

**Figure 4:**
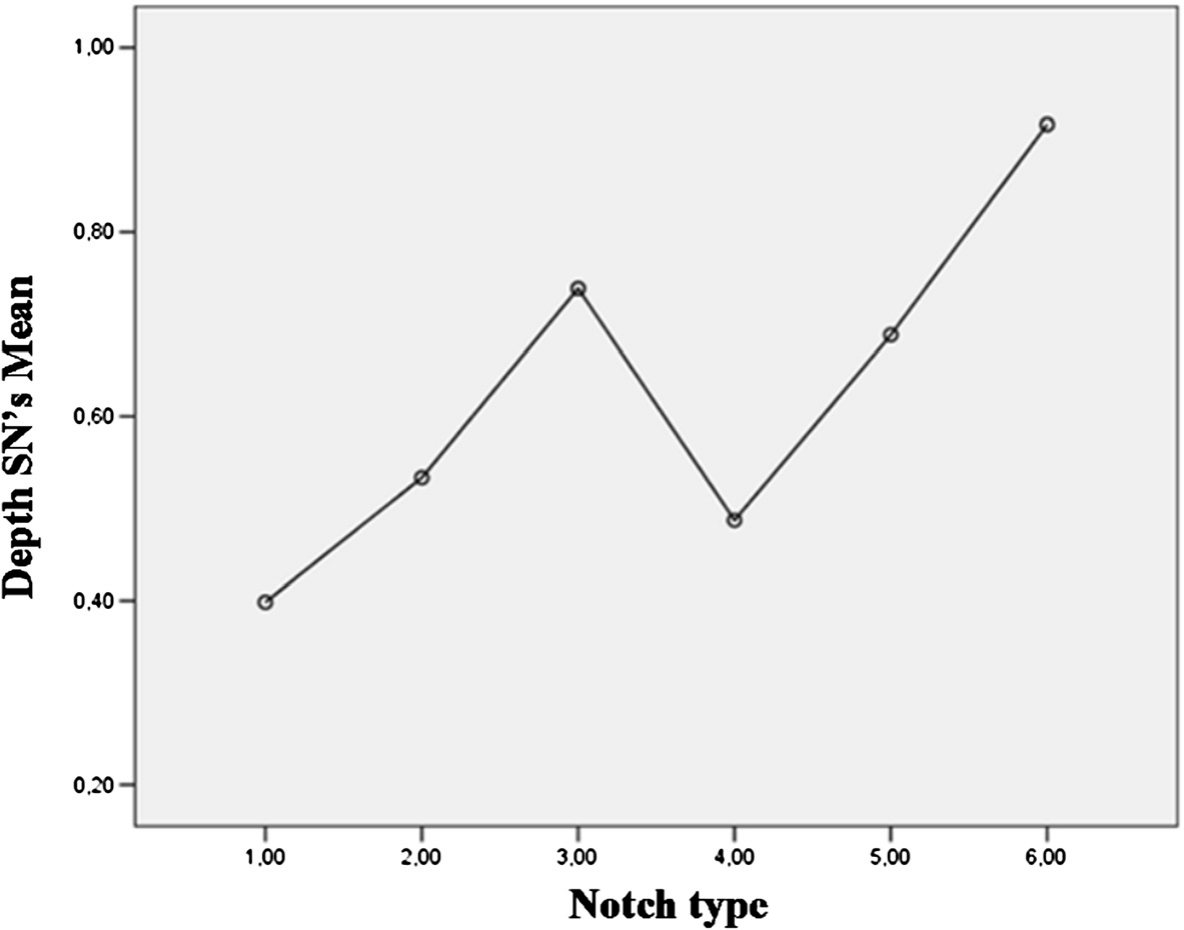
**Relationship between notch type and depth of the suprascapular notch (mean).** SN= suprascapular notch.

**Figure 5:**
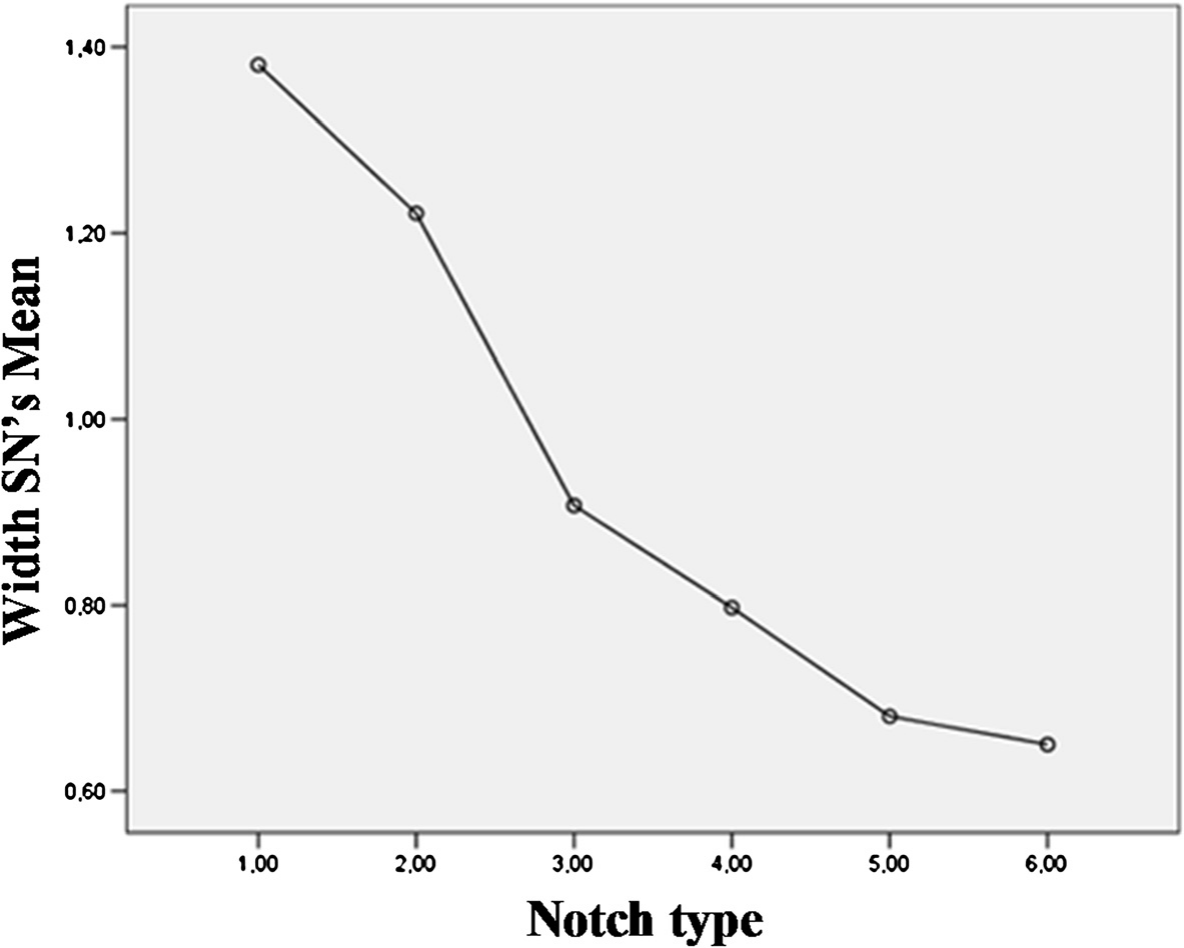
**Relationship between notch type and width of the suprascapular notch (mean).** SN= suprascapular notch.

Analyzing the relationship between dimensions and types of the suprascapular notch, we found a statistically significant difference (p< 0.0001) in depth between Type IV and the last two types (V,VI), between Type III and Type IV, between Type II and Type III, V and VI as well as between Type I and each of the remaining types. We also found a significant difference (p< 0.0001) in width between Type IV and Type V, between Type III and Type IV, between V and VI, between Type II and Type III, IV, V and VI as well as between Type I and each of the remaining types. Figures 4 and 5 show such trends. Table 4 describes the specific values of the SN dimensions for each notch type.

**Table 4:** Descriptive analysis of the dimensions of the suprascapular notch types

	**Mean (cm)**	**Std. deviation**	**Minimum (cm)**	**Maximum (cm)**	**Median (cm)**
**Type I**					
**D SN**	0.39	0.17	0.10	1.00	0.40
**W SN**	1.38	0.37	0.50	2.20	1.40
**Type II**					
**D SN**	0.53	0.15	0.20	1.00	0.50
**W SN**	1.22	0.36	0.50	3.10	1.20
**Type III**					
**D SN**	0.73	0.22	0.20	1.60	0.70
**W SN**	0.90	0.24	0.40	1.70	0.90
**Type IV**					
**D SN**	0.48	0.15	0.10	1.10	0.50
**W SN**	0.79	0.22	0.30	1.40	0.80
**Type V**					
**D SN**	0.68	0.23	0.30	1.40	0.70
**W SN**	0.68	0.22	0.10	1.30	0.70
**Type VI**					
**D SN**	0.91	0.37	0.20	1.90	0.90
**W SN**	0.65	0.22	0.40	1.10	0.60

*DSN* depth of the suprascapular notch.

*WSN* width of the suprascapular notch.

**Figure 6:**
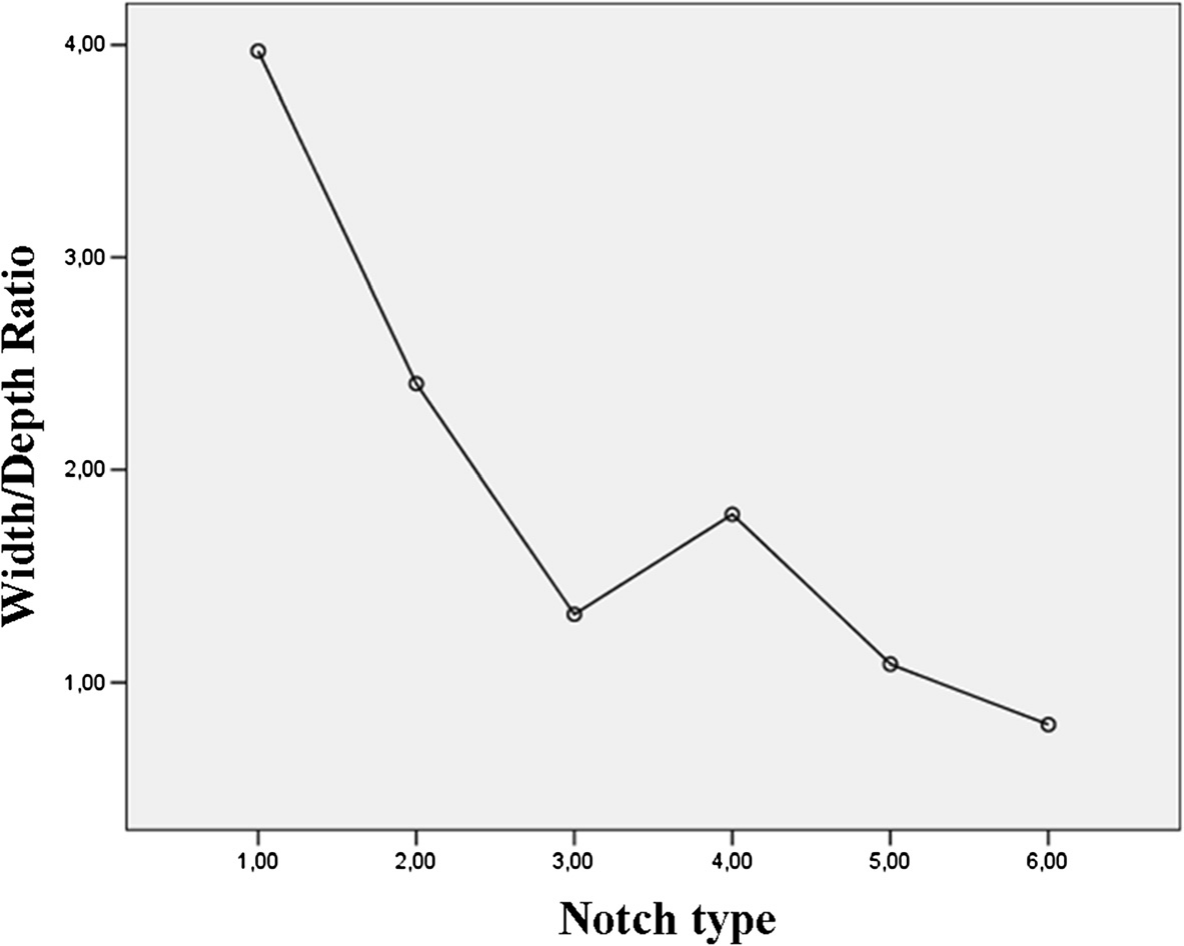
Relationship between width/depth ratio and SN types.

The analysis of the width/depth ratio showed a statistically significant difference between all notch types except between Type III and Type V and between Type V and Type VI. The highest ratio was associated to Type I (3.97 cm; IC 95%: 3.50-4.43 cm). This is followed respectively in descending order, by Types II, IV, III and V. The lowest ratio belonged to Type VI (0.80 cm; IC 95%: 0.60-0.99 cm). Details are found in Figure 6.

**Figure 7:**
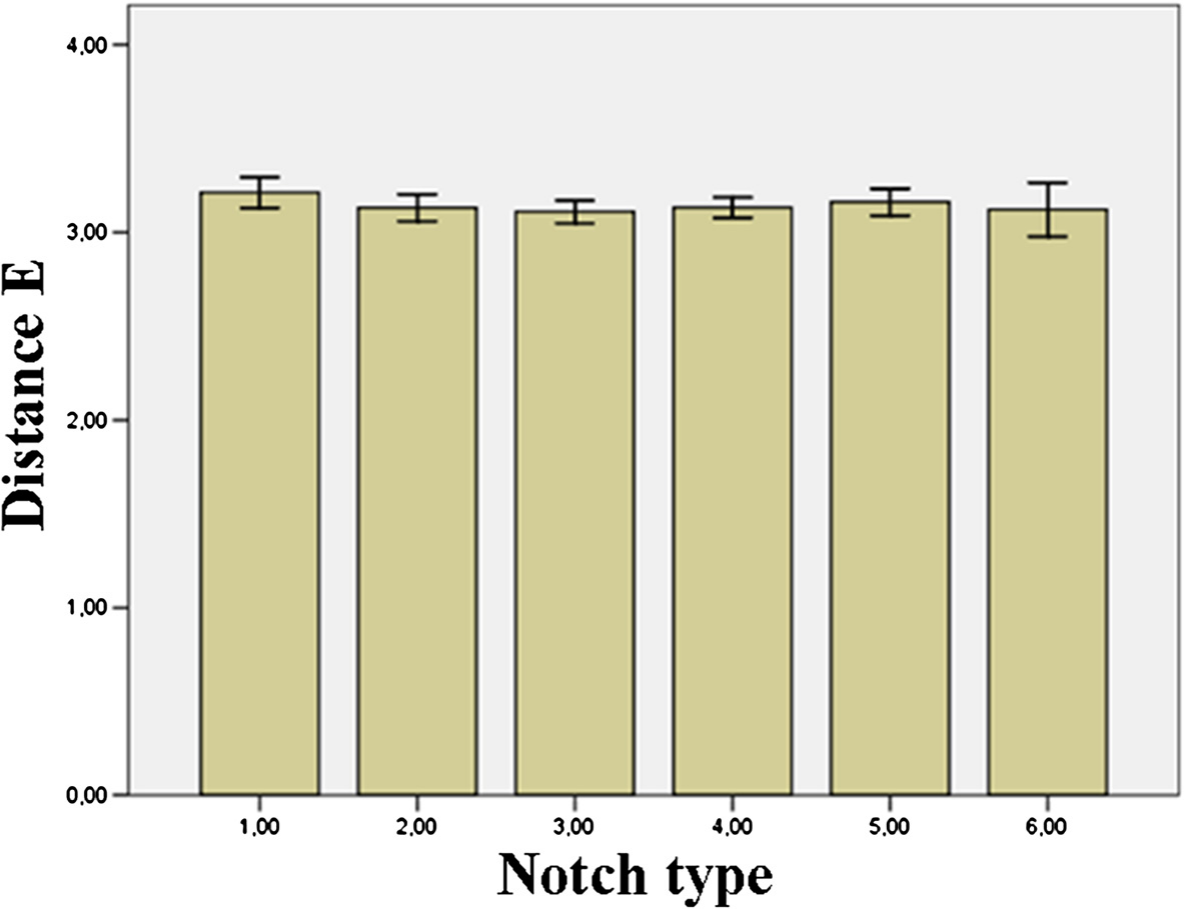
Relationship between distance E (mean) and the six notch types.

Finally, there was a not statistically significant difference between the posterosuperior limit of the safe zone (distance E) and the six types of notches. These findings are summarized in Figure 7.

The intraclass correlation coefficient which assesses the error of the vernier calliper was 0.996 with a 95% Confidence Interval: 0.993-0.998.

## Discussion

In the relevant scientific literature, we found many studies [3,9,10,12,28-32] that investigated the characteristics of the suprascapular notch; unfortunately there is not a univocal standard of classification [3,10,33] whereupon the opportunity to compare the different results is reduced.

In fact, taking the cue from Rengachary's study [3], other authors elaborated the geometric and morphologic features of the notches in order to create and refine new models of classification. In 2007 Nastis et al. [10] presented their classification, distinguishing V classes on the base of the relationship between the vertical and transverse diameter of the SSN and the presence/absence of both notch and foramina. A further system of classification was elaborated by Polguj et al. in [33]. They compared for every single notch 3 distances: the maximal depth, the superior and middle transverse diameter; successively they divided the notches in V main classes basing on morphologic features, with a further division 3 in sub-classes for both type I and III, according to the specific relationship between the 3 distances considered. Although these systems may seem more specific if compared with Rengachary's classification [3] they are quite new, they are not yet fully assessed and they own a low diffusion at the time.

Considering those studies conducted on dry scapulae and adopting the Rengachary’s system of classification [3,31], we observed a difference related to the frequencies of the SN Types. In fact, previously Type III was considered the most common class while Type IV represented only 3-5% of the population. In our study Type IV resulted the most common class and its frequency proved to be six to ten times higher than formerly reported (Table 5). Although the differences might be influenced by the racial characteristics of the donors, we believe this discrepancy is due to the significantly lower number of scapulae analyzed in other studies. The incidence of complete ossification of the superior transverse scapular ligament was similar among considered studies, varying from 3.6% to 4%; the same data in the whole literature varies from 3.7% to 12.5% [3,9,12,28,29,34-36]. Although the differences might be influenced by the different samples analyzed and/or methods of evaluation and classification, we agree with the assumption that the occurrence of a complete ossification of the STSL could have basically a genetic influence [33]. In fact, the frequency of the foramina showed very different throughout the world, Hrdicka [37] found 0.2% in the Alaskan Eskimo, turkish population presents a 12.5% of foramina [12], Khan [38] found only one case in India. Furthermore, Cohen et al. [11] described a familiar case of calcification of the STSL affecting a 58-year-old man and his son causing suprascapular nerve entrapment syndrome with a symptomatic involvement of the supraspinatus muscle.

**Table 5:** Comparison of the percentages of the different types of suprascapular notch presented in literature

	**Type I**	**Type II**	**Type III**	**Type IV**	**Type V**	**Type VI**	**N**
**Rengachary et al.**	8%	31%	48%	3%	6%	4%	211
**Sinkeet et al.**	22%	21%	29%	5%	18%	4%	135
**Our study**	12.4%	19.8%	22.8%	31.1%	10.2%	3.6%	500

*N* number of the sample.

We believe that the difference between the mean depths, elaborated according to our median age, may be due to the most frequent presence of partial/total ossification of the superior transverse scapular ligament in the population over 73 years old. At the same time, we are aware that our sample, which is made up of dry scapulae from cadavers, is not homogeneous in terms of age (95.8% of the sample was over 50 years old). Therefore it is worth further investigating this assumption.

The correlation indexes developed in this study showed a weak relationship between the dimensions of the suprascapular notch and the glenoid fossa, while the relationship between the dimensions of the suprascapular notch and the scapular body was absent. The strongest correlation was found between the posterosuperior limit of the safe zone and the width of the suprascapular notch. Unfortunately this relationship may be facilitated by a geometric element. Both distances share a little segment of the scapula (from the lateral angle of the suprascapular notch to the middle part of the notch itself) and we suppose that the overlapping may affect the results. As far as our analysis is concerned, we can state that the dimensions of the scapula could not be considered as predictive parameters to determine the dimensions of the suprascapular notch; those should be considered independent variables.

Recently, Polguj et al. [33] analyzed a sample of 86 dried scapulae and demonstrated the existence of a direct correlation between the scapular length (corresponding to A axis in our study) and the SN depth (R=0.265) and an inverse correlation between the ratio length/width of the scapular body and the SN depth (R=−0.327). In our opinion, the different statistical method used to determine the correlation (Spearman’s rank correlation coefficient) as well as the size of the sample considered may have led to a discrepancy in the results of the two works.

The dimensions of the suprascapular notch were compared with those presented in previous studies [3,9,32] (Table 6); although the ranges of both distances proved to be wider than formerly assumed, we found the averages presented by the other authors (elaborated both for the whole population and for each notch type) to be over-estimated. However, our findings highlighted the great variability in the notch dimensions that can be found among the whole population and ,at the same time, demonstrated that the notch dimensions are averagely shorter than previously assumed; consequently the area available for the suprascapular nerve should be expected to be smaller too.

**Table 6:** Comparison of the averages and ranges of the scapular notch dimensions presented in literature

	**Average ± SD (cm)**		**Range min-max (cm)**		**N**
	**W**	**D**	**W**	**D**	
**Rengachary et al.**					211
**Type II**	1.67±0.51	0.97±0.35	/	/	
**Type III**	1.01±0.27	1.12±0.37	/	/	
**Type IV**	0.81±0.22	0.93±0.47	/	/	
**Thicker et al.**					79
**WP**	/	/	0.5-1.7	0.4-1.2	
**Wang et al.**					295
**WP**	1.29±0.41	0.71±0.27	/	/	
**Our study**					500
**WP**	0.96±0.36	0.57±0.23	0.10-1.90	0.10-3.10	
**Type II**	1.22±0.36	0.53±0.15	0.50-3.10	0.20-1.00	
**Type III**	0.90±0.24	0.73±0.22	0.40-1.70	0.20-1.60	
**Type IV**	0.79±0.22	0.48±0.15	0.30-1.40	0.10-1.10	

*SD* standard deviation, *W* width; *D* depth.

*WP* whole population, *N* number of the sample.

In analyzing the trends reported in this study, we found that Type III, V and VI have the lowest width/depth ratio (indicative of the area occupied by the suprascapular notch). Dunkelgrun et al. [36] stated that type III notches had a larger area than Type IV notches, leading to the assumption that a V-shaped notch would be more likely connected with nerve entrapment. At the same time, Rengachary et al. [3] concluded their work asserting that patients with Type IV notch are more likely to suffer from the suprascapular nerve entrapment syndrome; but in clinical practice, because of the higher frequency of Type III scapulae and its associated small size, it is more likely to encounter an entrapment syndrome associated with this type of notch. We agree with Regngachary’s assumption [3] that patients with Type III notch are more likely to have suprascapular entrapment neuropathy than patients with Type IV notch. But we believe that the main pattern that predisposes patients to the entrapment syndrome involves the specific anatomical characteristics of Type III notches instead of the frequency, which proved to be almost nine percentage points higher for Type IV. In fact, comparing the width/depth ratio between the two classes, we observed that Type III notch presents averagely a lower value and consequently a smaller space available for the suprascapular nerve, increasing the possibility to have a case of suprscapular nerve entrapment. Because our analysis oppose to those presented by Dunkelgrun et al. [36], we believe that the different aims and methods of the two studies may explain the difference in the results.

Sinkeet et al. [31] reported that Type III notches were associated with the lowest value of the posterosuperior limit (difference among such types are not statistically significant). Wang et al. [32] found that Type IV notches have the shortest posterosuperior limit and their conclusions were corroborated by the work of Urgüden et al. [30]. Our findings agree with Sinkeet et al’s work [31]. We have also demonstrated that the posterosuperior limit of the safe zone [27] does not change considerably from Type I to Type VI and, at the same time, the difference among them was not statistically significant.

One of the limits of our study is due to the sample composed of dried scapulae. In fact, the anatomical variations of the anterior coracoscapular ligament as well as the existence of a bifid or trifid STSL, that are important anatomical factors influencing the incidence of the suprascapular nerve entrapment syndrome [39-42] can only be evaluated in vivo. Further studies are required to investigate this specific issue.

## Conclusions

In conclusion, we believe that the characteristics of the patient (gender, age and dimensions of the scapula) are not related to the characteristics of the suprascapular notch (dimensions and Type). Our findings demonstrated that entrapment syndrome is more likely to be associated with a Type III notch because of its specific features.

## Competing interests

The authors declare that they have no competing interests.

## Authors’ contributions

PA; Examined and collected the data related to the anatomy of the scapulae. SC; Drafted and prepared the manuscript for submission. VC; Provided assistance in collecting the sample and examined the data related to the anatomy of the scapulae. VA; Provided assistance in collecting the sample and wrote the manuscript. ARV; Developed the statistical analysis. SG; Created and managed the development of each phase of the study. All authors reviewed and critically revised the manuscript drafts, and read and approved the final manuscript.

## Pre-publication history

The pre-publication history for this paper can be accessed here:

http://www.biomedcentral.com/1471-2474/14/172/prepub

## References

[B1] Williams PL Bannister LH Bery MM Gray's anatomy 1995 38 Edinburgh: Churchill Livingstone

[B2] Kopell HP Thompson WA Pain and the frozen shoulder Surg Gynecol Obstet 1959 109 92 96 13668845

[B3] Rengachary SS Neff JP Singer PA Brackett CF Suprascapular entrapment neuropathy. A clinical, anatomical and comparative study, Part I Neurosurgery 1979 4 441 446 534047 10.1227/00006123-197910000-00006

[B4] Shishido H Kikuchi S Injury of the suprascapular nerve in shoulder surgery: An anatomic study J Shoulder Elbow Surg 2001 10 372 376 10.1067/mse.2001.115988 11517368

[B5] Burkhart SS Lo IK Brady PC Burkhart’s view of the shoulder a cowboy’s guide to advanced shoulder arthroscopy 2006 Philadelphia: Lippincott, Williams & Williams 111 116 194-203

[B6] Antoniou J Tae SK Williams GR Bird S Ramsey ML Iannotti JP Suprascapular neuropathy. Variability in the diagnosis, treatment and outcome Clin Orthop 2001 386 131 138 11347826

[B7] Piatt BE Hawkins RJ Fritz RC Ho CP Wolf E Schickendantz M Clinical evaluation and treatment of spinoglenoid notch ganglion cysts J Shoulder Elbow Surg 2002 11 600 604 10.1067/mse.2002.127094 12469086

[B8] Hazrati Y Miller S Moore S Hausman M Flatow E Suprascapular nerve entrapment secondary to a lipoma Clin Orthop 2003 411 124 128 12782867 10.1097/01.blo.0000063791.32430.59

[B9] Ticker JB Djurasovic M Strauch RJ The incidence of ganglion cysts and other variations in anatomy along the course of the suprascapular nerve J Shoulder Elbow Surg 1998 7 472 478 10.1016/S1058-2746(98)90197-5 9814925

[B10] Natsis K Totlis T Tsikaras P Appell HJ Skandalakis P Koebke J Proposal for classification of the suprascapular notch: A study on 423 dried scapulas Clin Anat 2007 20 135 139 10.1002/ca.20318 16838269

[B11] Cohen SB Dines DM Moorman CT Familiar calcification of the superior transverse scapular ligament causing neuropathy Clin Orthop 1997 131 135 9005905

[B12] Bayramoglu A Demiryürek D Tüccar E Variations in anatomy at the suprascapular notch possibly causing suprascapular nerve entrapment: An anatomical study Knee Surg Sports Traumatol Arthrosc 2003 11 393 398 10.1007/s00167-003-0378-3 12830371

[B13] Shaffer BS Conway J Jobe FW Kvitne RS Tibone JE Infraspinatus muscle-splitting incision in posterior shoulder surgery Am J Sports Med 1994 22 113 120 http://dx.doi.org/10.1016/S1058-2746(02)00034-4 10.1177/036354659402200118 8129093

[B14] Costouros JG Porramatikul M Lie DT Warner JJP Reversal of suprascapular neuropathy following arthroscopic repair of massive supraspinatus and infraspinatus rotator cuff tears Arthroscopy 2007 11 1152 1161 10.1016/j.arthro.2007.06.014 17986401

[B15] Yoo JC Lee YS Ahn JH Park JH Kang HJ Koh KH Isolated suprascapular nerve injury below the spinoglenoid notch after SLAP repair J Shoulder Elbow Surg 2009 18 e27 e29 10.1016/j.jse.2008.10.006 19119021

[B16] Asami A Sonohata M Morisawa K Bilateral suprascapular nerve entrapment syndrome associated with rotator cuff tear J Shoulder Elbow Surg 2000 9 70 72 10.1016/S1058-2746(00)90013-2 10717866

[B17] Bittar ES Arthroscopic management of massive rotator cuff tears Arthroscopy 2002 9 104 106 10.1053/jars.2002.36512 12426534

[B18] Debeyre J Patte D Elmelik E Repair of ruptures of the rotator cuff of the shoulder- with a note on advancement of the supraspinatus muscle J Bone Joint Surg 1980 62A 897 908

[B19] Meyer M Graveleau N Hardy P Landreau P Anatomic risks of shoulder arthroscopy portals: anatomic cadaveric study of 12 portals Arthroscopy 2007 23 529 536 10.1016/j.arthro.2006.12.022 17478285

[B20] Neer CS Impingement lesions Clin Orthop 1983 173 70 77 6825348

[B21] Neri BR Chan KW Kwon YW Management of massive and irreparablerotator cuff tears J Shoulder Elbow Surg 2009 18 808 818 10.1016/j.jse.2009.03.013 19487132

[B22] Warner JJP Krushell RJ Masquelet A Gerber C Anatomy and relationships of the suprascapular nerve: anatomical constraints to mobilization of the supraspinatus and infraspinatus muscles in the management of massive rotator-cuff tears J Bone Joint Surg Am 1992 74 36 45 1734012

[B23] Greiner A Golser K Wambacher M Kralinger F Sperner G The course of the suprascapular nerve in the supraspinatus fossa and its vulnerability in muscle advancement J Shoulder Elbow Surg 2003 12 256 259 http://dx.doi.org/10.1016/S1058-2746(02)00034-4 10.1016/S1058-2746(02)00034-4 12851579

[B24] Post M Mayer J Suprascapular nerve entrapment, diagnosis and treatment Clin Orthop 1987 223 126 136 3652566

[B25] Bigliani LU Dasley RM McCann PD April EW An anatomical study of the suprascapular nerve Arthroscopy 1990 6 301 305 http://dx.doi.org/10.1016/0749-8063(90)90060-Q 10.1016/0749-8063(90)90060-Q 2264898

[B26] Ekin A Magden O Iche C Vastamäki M, Jalovaara P Anatomy and relationship of the suprascapular nerve in surgery of the shoulder Surgery of the shoulder 1995 New York: Elsevier 379 392

[B27] Gumina S Albino P Giaracuni M Vestri AR Ripani M Postacchini F The safe zone for avoiding suprascapular nerve injury during shoulder arthroscopy: An anatomical study on 500 dry scapulae J Shoulder Elbow Surg 2011 20 8 1317 1322 10.1016/j.jse.2011.01.033 21493105

[B28] Edelson JG Bony bridges and other variations of the suprascapular notch J Bone Joint Surg Br 1995 77 505 506 7744948

[B29] Prescher A Anatomical basics, variations, and degenerative changes of the shoulder joint and shoulder girdle Eur J Radiol 2000 35 88 102 10.1016/S0720-048X(00)00225-4 10963915

[B30] Urgüden M Ozdemir H Dönmez B Bilbasar H Oguz N Is there any effect of suprascapular notch type in iatrogenic suprascapular nerve lesions? An anatomical study Knee Surg Sports Traumatol Arthrosc 2004 12 241 245 10.1007/s00167-003-0442-z 14658033

[B31] Sinkeet SR Awori KO Odula PO Ogeng’o JA Mwachaka PM The suprascapular notch: its morphology and distance from the glenoid cavity in a Kenyan population Folia Morphol (Warsz) 2010 4 241 245 21120811

[B32] Wang HJ Chen C Wu LP Pan CQ Zhang WJ Li YK Variable morphology of the suprascapular notch: an investigation and quantitative measurements in chinese population Clin Anat 2010 24 47 55 20890970 10.1002/ca.21061

[B33] Polguj M Jędrzejewski KS Podgórski M Topol M Correlation between morphometry of the suprascapular notch and anthropometric measurements of the scapula Folia Morphol 2011 70 109 115 21630232

[B34] Tubbs RS Smyth MD Salter G Oakes WJ Anomalous traversement of the suprascapular artery through the suprascapular notch: a possible mechanism for undiagnosed shoulder pain? Med Sci Monit 2003 9 116 119 12640333

[B35] Olivier G Pratique anthropologique. Le scapulum 1960 Paris: Vigot Freres 194 201

[B36] Dunkelgrun M Iesaka K Park SS Kummer FJ Zuckkerman JD Interobserver reliability and intraobserver reproducibility in suprascapular notch typing Bull Hosp Joint Dis 2003 61 118 122 15156809

[B37] Hrdicka A The adult scapula: visual observations Am J Phys Anthropol 1942 29 73 94 10.1002/ajpa.1330290107

[B38] Khan MA Complete ossification of the superior transverse scapular ligament in an Indian male adult Int J Morphol 2006 24 195 196

[B39] Avery BW Pilon FM Barclay JK Anterior coracoscapular ligament and suprascapular nerve entrapment Clin Anat 2002 15 383 386 10.1002/ca.10058 12373728

[B40] Zehetgruber H Noske H Lang T Wurnig C Suprascapular nerve entrapment A meta-analysis. Int Orthop 2002 26 339 343 10.1007/s00264-002-0392-y PMC3620977 12466865

[B41] Duparc F Coquerel D Ozeel J Noyon M Gerometta A Michot C Anatomical basis of the suprascapular nerve entrapment and clinical relevance of the supraspinatus fascia Surg Rad Anat 2010 32 277 284 10.1007/s00276-010-0631-7 20309668

[B42] Polguj M Jedrzejewski KS Majos A Topol M The trifid superior transverse scapular ligament: a case report and review of the literature Folia Morph (Warsz.) 2012 71 2 118 120 22648592

